# 3-Hy­droxy-2-[(4-hy­droxy-3,5-dimeth­oxy­phen­yl)(2-hy­droxy-4,4-dimethyl-6-oxo­cyclo­hex-1-en-1-yl)meth­yl]-5,5-dimethyl­cyclo­hex-2-en-1-one

**DOI:** 10.1107/S1600536811002698

**Published:** 2011-01-26

**Authors:** Xiao-Hui Yang, Yong-Hong Zhou, Meng Zhang, Li-Hong Hu

**Affiliations:** a Institute of Chemical Industry of Forest Products, Chinese Academy of Forestry, Nanjing 210042, People’s Republic of China

## Abstract

In the title compound, C_25_H_32_O_7_, the 3-hy­droxy-5,5-dimethyl­cyclo­hex-2-enone rings adopt slightly distorted envelope conformations with the two planes at the base of the envelope forming dihedral angles of 57.6 (4) and 53.9 (9)° with the benzene ring. There is an intra­molecular hy­droxy–ketone O—H⋯O inter­action between the two substituted cyclo­hexane rings as well as a short intra­molecular phenol–meth­oxy O—H⋯O inter­action.

## Related literature

For related structures, see: Yang *et al.* (2010 ▶); Tu *et al.* (2004 ▶). For applications of 1,4-dihydro­pyridine derivatives, see: Rose & Draeger (1992 ▶); Davies *et al.* (2005 ▶); Warrior *et al.* (2005 ▶).
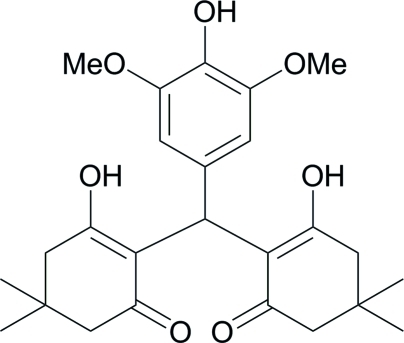


## Experimental

### 

#### Crystal data

C_25_H_32_O_7_
*M* *_r_* = 444.51
Triclinic, 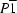

*a* = 9.1620 (18) Å
*b* = 10.979 (2) Å
*c* = 13.120 (3) Å
α = 100.82 (3)°
β = 109.04 (3)°
γ = 104.16 (3)°
*V* = 1157.0 (6) Å^3^
*Z* = 2
Mo *K*α radiation
μ = 0.09 mm^−1^
*T* = 293 K
0.30 × 0.20 × 0.20 mm

#### Data collection

Enraf–Nonius CAD-4 diffractometer
Absorption correction: ψ scan (North *et al.*, 1968 ▶) *T*_min_ = 0.973, *T*_max_ = 0.982
4515 measured reflections
4230 independent reflections
2652 reflections with *I* > 2σ(*I*)
*R*_int_ = 0.023
3 standard reflections every 200 reflections  intensity decay: 1%

#### Refinement

*R*[*F* ^2^ > 2σ(*F* ^2^)] = 0.059
*wR*(*F* ^2^) = 0.184
*S* = 1.00
4230 reflections
289 parameters
H-atom parameters constrained
Δρ_max_ = 0.16 e Å^−3^
Δρ_min_ = −0.30 e Å^−3^

### 

Data collection: *CAD-4 EXPRESS* (Enraf–Nonius, 1994 ▶); cell refinement: *CAD-4 EXPRESS*; data reduction: *XCAD4* (Harms & Wocadlo, 1996 ▶); program(s) used to solve structure: *SHELXS97* (Sheldrick, 2008 ▶); program(s) used to refine structure: *SHELXL97* (Sheldrick, 2008 ▶); molecular graphics: *SHELXTL* (Sheldrick, 2008 ▶); software used to prepare material for publication: *SHELXTL*.

## Supplementary Material

Crystal structure: contains datablocks I, global. DOI: 10.1107/S1600536811002698/zs2081sup1.cif

Structure factors: contains datablocks I. DOI: 10.1107/S1600536811002698/zs2081Isup2.hkl

Additional supplementary materials:  crystallographic information; 3D view; checkCIF report

## Figures and Tables

**Table 1:** Hydrogen-bond geometry (Å, °)

*D*—H⋯*A*	*D*—H	H⋯*A*	*D*⋯*A*	*D*—H⋯*A*
O5—H5*B*⋯O6	0.82	1.80	2.604 (3)	166
O7—H7*D*⋯O4	0.82	1.84	2.647 (3)	168

**Fig. 1.**
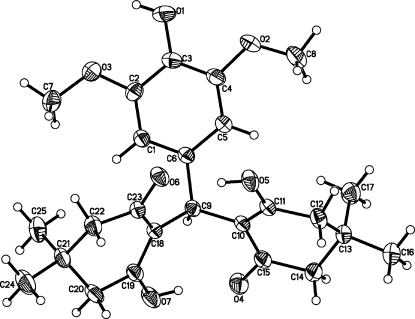
Molecular structure of the title compound, showing the atom-numbering scheme. Displacement ellipsoids are drawn at the 50% probability level.

## supplementary crystallographic information

### Comment

The development of new methods for the synthesis of 1,4-dihydropyridine derivatives is the motive for the current study, these derivatives being of interest because of their presence in numerous natural products and as well they possess a wide range physiological activities, e.g. they have calcium modulatory properties (Rose & Draeger, 1992), antibacterial activity (Davies *et al.*, 2005) and fungicidal activity (Warrior *et al.*, 2005). In order to prepare 1,4-dihydropyridine and research its bioactivity, the intermediate product C_25_H_32_O_7_, the title compound (I) was synthesized and its crystal structure is presented here. In the molecular structure of (I) (Fig. 1), the two fused 3-hydroxy-5,5-dimethylcyclohex-2-enone rings can be regarded as having envelope conformations, with atom C13 0.66 (6) Å out of the plane of atoms C10/C11/C12/C14/C15 and atom C21 0.65 (5) Å out of the plane of atoms C18/C19/C20/C22/C23. In addition, the two planes form dihedral angles of 57.6 (4)° and 53.9 (9)° with the phenyl ring. The methoxy group is nearly coplanar with the attached benzene ring [torsion angle C8—O2—C4—C5, -2.4 (5)°]. The crystal packing of the title compound is stabilized by two intra- cyclohexane ring hydroxy O—H···O_ketone_ hydrogen bonds and a single intramolecular phenol O—H···O3_methoxy_ interaction (Table 1).

### Experimental

A mixture of 4-hydroxy-3,5-dimethoxybenzaldehyde (2 mmol) and 4-hydroxy-3,5-dimethoxy (4 mmol) was stirred in water (2 ml) at 353 K. After completion of the reaction (TLC monitoring), the mixture was diluted with cold water (20 ml) and filtered to obtain the precipitated product which was further purified by recrystallization. Single crystals suitable for X-ray diffraction were obtained by slow evaporation of an ethanol solution.

### Refinement

The H atoms were fixed geometrically and allowed to ride on the attached non-H atoms, with O—H = 0.82–0.85 Å and C—H = 0.93–0.97 Å, and with *U*_iso_(H) = 1.5 *U*_eq_(C) for methyl H atoms and 1.2 *U*_eq_(C) for all other atoms.

### Crystal data

**Table:**

C_25_H_32_O_7_	*Z* = 2
*M**_r_* = 444.51	*F*(000) = 476
Triclinic, *P*1	*D*_x_ = 1.276 Mg m^−^^3^
Hall symbol: -P 1	Mo *K*α radiation, λ = 0.71073 Å
*a* = 9.1620 (18) Å	Cell parameters from 25 reflections
*b* = 10.979 (2) Å	θ = 8–14°
*c* = 13.120 (3) Å	µ = 0.09 mm^−^^1^
α = 100.82 (3)°	*T* = 293 K
β = 109.04 (3)°	Block, colourless
γ = 104.16 (3)°	0.30 × 0.20 × 0.20 mm
*V* = 1157.0 (6) Å^3^	

### Data collection

**Table:**

Enraf–Nonius CAD-4 diffractometer	2652 reflections with *I* > 2σ(*I*)
Radiation source: fine-focus sealed tube	*R*_int_ = 0.023
graphite	θ_max_ = 25.4°, θ_min_ = 1.7°
ω/–2θ scans	*h* = 0→11
Absorption correction: ψ scan (North *et al.*, 1968)	*k* = −13→12
*T*_min_ = 0.973, *T*_max_ = 0.982	*l* = −15→14
4515 measured reflections	3 standard reflections every 200 reflections
4230 independent reflections	intensity decay: 1%

### Refinement

**Table:**

Refinement on *F*^2^	Primary atom site location: structure-invariant direct methods
Least-squares matrix: full	Secondary atom site location: difference Fourier map
*R*[*F*^2^ > 2σ(*F*^2^)] = 0.059	Hydrogen site location: inferred from neighbouring sites
*wR*(*F*^2^) = 0.184	H-atom parameters constrained
*S* = 1.00	*w* = 1/[σ^2^(*F*_o_^2^) + (0.1*P*)^2^ + 0.18*P*] where *P* = (*F*_o_^2^ + 2*F*_c_^2^)/3
4230 reflections	(Δ/σ)_max_ < 0.001
289 parameters	Δρ_max_ = 0.16 e Å^−^^3^
0 restraints	Δρ_min_ = −0.30 e Å^−^^3^

### Special details

**Table:**

Geometry. All esds (except the esd in the dihedral angle between two l.s. planes) are estimated using the full covariance matrix. The cell esds are taken into account individually in the estimation of esds in distances, angles and torsion angles; correlations between esds in cell parameters are only used when they are defined by crystal symmetry. An approximate (isotropic) treatment of cell esds is used for estimating esds involving l.s. planes.
Refinement. Refinement of *F*^2^ against ALL reflections. The weighted *R*-factor *wR* and goodness of fit *S* are based on *F*^2^, conventional *R*-factors *R* are based on *F*, with *F* set to zero for negative *F*^2^. The threshold expression of *F*^2^ > σ(*F*^2^) is used only for calculating *R*-factors(gt) *etc*. and is not relevant to the choice of reflections for refinement. *R*-factors based on *F*^2^ are statistically about twice as large as those based on *F*, and *R*- factors based on ALL data will be even larger.

### Fractional atomic coordinates and isotropic or equivalent isotropic displacement parameters (Å^2^)

**Table:**

	*x*	*y*	*z*	*U*_iso_*/*U*_eq_	
C1	0.3616 (3)	0.2008 (3)	0.8305 (2)	0.0423 (7)	
H1A	0.4668	0.2361	0.8339	0.051*	
O1	0.1583 (3)	0.0314 (2)	0.98624 (19)	0.0696 (7)	
H1B	0.2420	0.0296	1.0380	0.084*	
C2	0.3382 (3)	0.1413 (3)	0.9100 (2)	0.0454 (7)	
O2	−0.0996 (3)	0.0423 (2)	0.82596 (19)	0.0702 (7)	
O3	0.4598 (3)	0.1312 (2)	0.99870 (18)	0.0603 (6)	
C3	0.1799 (4)	0.0880 (3)	0.9058 (2)	0.0486 (7)	
O4	0.1863 (2)	0.1972 (2)	0.42127 (16)	0.0618 (6)	
C4	0.0503 (3)	0.0955 (3)	0.8216 (2)	0.0490 (7)	
O5	0.0426 (2)	0.4150 (2)	0.69521 (17)	0.0542 (6)	
H5B	0.1400	0.4341	0.7342	0.081*	
C5	0.0728 (3)	0.1532 (3)	0.7405 (2)	0.0464 (7)	
H5A	−0.0172	0.1550	0.6823	0.056*	
O6	0.3409 (2)	0.49561 (19)	0.84537 (16)	0.0516 (5)	
C6	0.2298 (3)	0.2087 (2)	0.7456 (2)	0.0383 (6)	
O7	0.4945 (2)	0.3343 (2)	0.55456 (17)	0.0652 (7)	
H7D	0.3958	0.2967	0.5204	0.098*	
C7	0.6237 (4)	0.1861 (4)	1.0097 (3)	0.0652 (9)	
H7A	0.6968	0.1721	1.0744	0.098*	
H7B	0.6486	0.2787	1.0189	0.098*	
H7C	0.6362	0.1447	0.9433	0.098*	
C8	−0.2392 (4)	0.0506 (4)	0.7457 (3)	0.0733 (10)	
H8A	−0.3340	0.0092	0.7586	0.110*	
H8B	−0.2507	0.0070	0.6715	0.110*	
H8C	−0.2284	0.1412	0.7521	0.110*	
C9	0.2629 (3)	0.2702 (2)	0.6556 (2)	0.0363 (6)	
H9A	0.2940	0.2056	0.6123	0.044*	
C10	0.1132 (3)	0.2811 (3)	0.5686 (2)	0.0378 (6)	
C11	0.0111 (3)	0.3455 (3)	0.5945 (2)	0.0401 (6)	
C12	−0.1474 (3)	0.3377 (3)	0.5066 (3)	0.0500 (7)	
H12A	−0.1317	0.4185	0.4855	0.060*	
H12B	−0.2283	0.3320	0.5398	0.060*	
C13	−0.2147 (3)	0.2233 (3)	0.4010 (2)	0.0475 (7)	
C14	−0.0754 (3)	0.2221 (3)	0.3619 (2)	0.0578 (8)	
H14A	−0.1097	0.1414	0.3020	0.069*	
H14B	−0.0545	0.2947	0.3306	0.069*	
C15	0.0812 (3)	0.2323 (3)	0.4535 (2)	0.0461 (7)	
C16	−0.3548 (4)	0.2419 (4)	0.3101 (3)	0.0692 (10)	
H16A	−0.4423	0.2417	0.3352	0.104*	
H16B	−0.3937	0.1715	0.2420	0.104*	
H16C	−0.3166	0.3241	0.2961	0.104*	
C17	−0.2806 (4)	0.0945 (3)	0.4246 (3)	0.0622 (9)	
H17A	−0.1942	0.0812	0.4818	0.093*	
H17B	−0.3222	0.0231	0.3568	0.093*	
H17C	−0.3670	0.0981	0.4500	0.093*	
C18	0.4135 (3)	0.3933 (3)	0.7051 (2)	0.0374 (6)	
C19	0.5225 (3)	0.4114 (3)	0.6525 (2)	0.0460 (7)	
C20	0.6797 (3)	0.5237 (3)	0.7034 (3)	0.0550 (8)	
H20A	0.6617	0.5971	0.6760	0.066*	
H20B	0.7593	0.4981	0.6785	0.066*	
C21	0.7493 (3)	0.5682 (3)	0.8309 (2)	0.0505 (7)	
C22	0.6138 (3)	0.5941 (3)	0.8669 (2)	0.0510 (7)	
H22A	0.6463	0.6024	0.9467	0.061*	
H22B	0.6044	0.6775	0.8566	0.061*	
C23	0.4487 (3)	0.4906 (3)	0.8044 (2)	0.0402 (6)	
C24	0.8977 (4)	0.6928 (4)	0.8753 (3)	0.0769 (11)	
H24A	0.8658	0.7605	0.8463	0.115*	
H24B	0.9806	0.6748	0.8513	0.115*	
H24C	0.9400	0.7214	0.9562	0.115*	
C25	0.8023 (4)	0.4613 (4)	0.8764 (3)	0.0732 (10)	
H25A	0.7096	0.3828	0.8496	0.110*	
H25B	0.8466	0.4903	0.9573	0.110*	
H25C	0.8841	0.4436	0.8510	0.110*	

### Atomic displacement parameters (Å^2^)

**Table:**

	*U*^11^	*U*^22^	*U*^33^	*U*^12^	*U*^13^	*U*^23^
C1	0.0363 (15)	0.0453 (16)	0.0461 (16)	0.0129 (13)	0.0173 (13)	0.0140 (13)
O1	0.0773 (16)	0.0842 (17)	0.0682 (15)	0.0278 (13)	0.0394 (13)	0.0476 (13)
C2	0.0482 (17)	0.0440 (16)	0.0440 (16)	0.0187 (14)	0.0141 (14)	0.0154 (13)
O2	0.0490 (13)	0.0912 (17)	0.0726 (15)	0.0050 (12)	0.0324 (12)	0.0372 (13)
O3	0.0537 (13)	0.0724 (15)	0.0626 (14)	0.0253 (11)	0.0205 (11)	0.0351 (12)
C3	0.0553 (19)	0.0460 (17)	0.0496 (17)	0.0118 (14)	0.0282 (15)	0.0180 (14)
O4	0.0482 (12)	0.0898 (16)	0.0484 (12)	0.0274 (12)	0.0249 (10)	0.0055 (11)
C4	0.0447 (17)	0.0519 (17)	0.0526 (17)	0.0092 (14)	0.0266 (15)	0.0154 (14)
O5	0.0387 (11)	0.0639 (13)	0.0556 (13)	0.0193 (10)	0.0203 (10)	0.0010 (10)
C5	0.0372 (15)	0.0557 (17)	0.0460 (16)	0.0108 (13)	0.0181 (13)	0.0168 (14)
O6	0.0428 (11)	0.0597 (13)	0.0539 (12)	0.0165 (10)	0.0272 (10)	0.0060 (10)
C6	0.0364 (15)	0.0387 (14)	0.0409 (15)	0.0111 (12)	0.0186 (12)	0.0097 (12)
O7	0.0413 (12)	0.0888 (17)	0.0559 (13)	0.0097 (11)	0.0277 (10)	−0.0002 (12)
C7	0.054 (2)	0.087 (2)	0.064 (2)	0.0362 (19)	0.0194 (17)	0.0315 (19)
C8	0.0450 (19)	0.091 (3)	0.083 (3)	0.0118 (18)	0.0328 (19)	0.024 (2)
C9	0.0324 (14)	0.0422 (15)	0.0387 (14)	0.0162 (12)	0.0174 (12)	0.0099 (12)
C10	0.0305 (14)	0.0439 (15)	0.0431 (15)	0.0120 (12)	0.0186 (12)	0.0148 (12)
C11	0.0322 (14)	0.0437 (15)	0.0466 (16)	0.0117 (12)	0.0189 (12)	0.0127 (13)
C12	0.0357 (15)	0.0515 (17)	0.0642 (19)	0.0172 (14)	0.0188 (14)	0.0172 (15)
C13	0.0354 (15)	0.0573 (18)	0.0494 (17)	0.0161 (14)	0.0132 (13)	0.0192 (14)
C14	0.0445 (18)	0.085 (2)	0.0428 (17)	0.0208 (17)	0.0160 (14)	0.0188 (16)
C15	0.0387 (16)	0.0570 (18)	0.0464 (17)	0.0164 (14)	0.0217 (13)	0.0134 (14)
C16	0.0461 (19)	0.090 (3)	0.068 (2)	0.0229 (18)	0.0134 (17)	0.033 (2)
C17	0.0524 (19)	0.057 (2)	0.062 (2)	0.0079 (16)	0.0129 (16)	0.0144 (16)
C18	0.0323 (14)	0.0434 (15)	0.0396 (14)	0.0130 (12)	0.0160 (12)	0.0147 (12)
C19	0.0359 (15)	0.0565 (18)	0.0455 (16)	0.0131 (13)	0.0198 (13)	0.0107 (14)
C20	0.0385 (16)	0.0622 (19)	0.064 (2)	0.0071 (14)	0.0298 (15)	0.0131 (15)
C21	0.0308 (15)	0.0565 (18)	0.0564 (18)	0.0072 (13)	0.0160 (14)	0.0101 (15)
C22	0.0400 (16)	0.0530 (18)	0.0504 (17)	0.0098 (14)	0.0152 (14)	0.0057 (14)
C23	0.0340 (15)	0.0446 (16)	0.0431 (15)	0.0135 (12)	0.0149 (12)	0.0143 (13)
C24	0.0440 (19)	0.077 (2)	0.086 (3)	−0.0034 (17)	0.0236 (18)	0.005 (2)
C25	0.0425 (19)	0.090 (3)	0.085 (3)	0.0297 (18)	0.0168 (18)	0.024 (2)

### Geometric parameters (Å, °)

**Table:**

C1—C2	1.376 (4)	C12—C13	1.513 (4)
C1—C6	1.384 (4)	C12—H12A	0.9700
C1—H1A	0.9300	C12—H12B	0.9700
O1—C3	1.365 (3)	C13—C14	1.525 (4)
O1—H1B	0.8500	C13—C17	1.526 (4)
C2—O3	1.368 (3)	C13—C16	1.527 (4)
C2—C3	1.403 (4)	C14—C15	1.504 (4)
O2—C4	1.379 (3)	C14—H14A	0.9700
O2—C8	1.402 (4)	C14—H14B	0.9700
O3—C7	1.422 (4)	C16—H16A	0.9600
C3—C4	1.365 (4)	C16—H16B	0.9600
O4—C15	1.280 (3)	C16—H16C	0.9600
C4—C5	1.381 (4)	C17—H17A	0.9600
O5—C11	1.295 (3)	C17—H17B	0.9600
O5—H5B	0.8200	C17—H17C	0.9600
C5—C6	1.392 (4)	C18—C19	1.385 (4)
C5—H5A	0.9300	C18—C23	1.406 (4)
O6—C23	1.276 (3)	C19—C20	1.495 (4)
C6—C9	1.542 (3)	C20—C21	1.513 (4)
O7—C19	1.305 (3)	C20—H20A	0.9700
O7—H7D	0.8200	C20—H20B	0.9700
C7—H7A	0.9600	C21—C25	1.523 (4)
C7—H7B	0.9600	C21—C24	1.528 (4)
C7—H7C	0.9600	C21—C22	1.533 (4)
C8—H8A	0.9600	C22—C23	1.501 (4)
C8—H8B	0.9600	C22—H22A	0.9700
C8—H8C	0.9600	C22—H22B	0.9700
C9—C10	1.519 (3)	C24—H24A	0.9600
C9—C18	1.525 (4)	C24—H24B	0.9600
C9—H9A	0.9800	C24—H24C	0.9600
C10—C11	1.389 (4)	C25—H25A	0.9600
C10—C15	1.409 (4)	C25—H25B	0.9600
C11—C12	1.503 (4)	C25—H25C	0.9600
			
C2—C1—C6	120.5 (3)	C15—C14—H14A	108.7
C2—C1—H1A	119.7	C13—C14—H14A	108.7
C6—C1—H1A	119.7	C15—C14—H14B	108.7
C3—O1—H1B	118.7	C13—C14—H14B	108.7
O3—C2—C1	125.3 (3)	H14A—C14—H14B	107.6
O3—C2—C3	114.6 (2)	O4—C15—C10	121.5 (3)
C1—C2—C3	120.2 (3)	O4—C15—C14	116.4 (2)
C4—O2—C8	118.6 (2)	C10—C15—C14	122.1 (2)
C2—O3—C7	117.5 (2)	C13—C16—H16A	109.5
O1—C3—C4	121.2 (3)	C13—C16—H16B	109.5
O1—C3—C2	119.7 (3)	H16A—C16—H16B	109.5
C4—C3—C2	119.1 (2)	C13—C16—H16C	109.5
C3—C4—O2	114.6 (2)	H16A—C16—H16C	109.5
C3—C4—C5	121.0 (3)	H16B—C16—H16C	109.5
O2—C4—C5	124.4 (3)	C13—C17—H17A	109.5
C11—O5—H5B	109.5	C13—C17—H17B	109.5
C4—C5—C6	120.1 (3)	H17A—C17—H17B	109.5
C4—C5—H5A	119.9	C13—C17—H17C	109.5
C6—C5—H5A	119.9	H17A—C17—H17C	109.5
C1—C6—C5	119.0 (2)	H17B—C17—H17C	109.5
C1—C6—C9	118.1 (2)	C19—C18—C23	117.7 (2)
C5—C6—C9	122.7 (2)	C19—C18—C9	119.8 (2)
C19—O7—H7D	109.5	C23—C18—C9	122.5 (2)
O3—C7—H7A	109.5	O7—C19—C18	122.9 (3)
O3—C7—H7B	109.5	O7—C19—C20	115.3 (2)
H7A—C7—H7B	109.5	C18—C19—C20	121.8 (3)
O3—C7—H7C	109.5	C19—C20—C21	113.5 (2)
H7A—C7—H7C	109.5	C19—C20—H20A	108.9
H7B—C7—H7C	109.5	C21—C20—H20A	108.9
O2—C8—H8A	109.5	C19—C20—H20B	108.9
O2—C8—H8B	109.5	C21—C20—H20B	108.9
H8A—C8—H8B	109.5	H20A—C20—H20B	107.7
O2—C8—H8C	109.5	C20—C21—C25	109.9 (3)
H8A—C8—H8C	109.5	C20—C21—C24	109.9 (3)
H8B—C8—H8C	109.5	C25—C21—C24	108.8 (3)
C10—C9—C18	115.0 (2)	C20—C21—C22	107.2 (2)
C10—C9—C6	114.9 (2)	C25—C21—C22	110.2 (3)
C18—C9—C6	113.0 (2)	C24—C21—C22	110.8 (3)
C10—C9—H9A	104.0	C23—C22—C21	115.0 (2)
C18—C9—H9A	104.0	C23—C22—H22A	108.5
C6—C9—H9A	104.0	C21—C22—H22A	108.5
C11—C10—C15	116.8 (2)	C23—C22—H22B	108.5
C11—C10—C9	124.4 (2)	C21—C22—H22B	108.5
C15—C10—C9	118.6 (2)	H22A—C22—H22B	107.5
O5—C11—C10	123.4 (2)	O6—C23—C18	121.5 (2)
O5—C11—C12	114.6 (2)	O6—C23—C22	116.7 (2)
C10—C11—C12	122.0 (2)	C18—C23—C22	121.8 (2)
C11—C12—C13	114.9 (2)	C21—C24—H24A	109.5
C11—C12—H12A	108.5	C21—C24—H24B	109.5
C13—C12—H12A	108.5	H24A—C24—H24B	109.5
C11—C12—H12B	108.5	C21—C24—H24C	109.5
C13—C12—H12B	108.5	H24A—C24—H24C	109.5
H12A—C12—H12B	107.5	H24B—C24—H24C	109.5
C12—C13—C14	107.1 (2)	C21—C25—H25A	109.5
C12—C13—C17	110.7 (2)	C21—C25—H25B	109.5
C14—C13—C17	111.1 (3)	H25A—C25—H25B	109.5
C12—C13—C16	109.6 (3)	C21—C25—H25C	109.5
C14—C13—C16	110.1 (2)	H25A—C25—H25C	109.5
C17—C13—C16	108.3 (2)	H25B—C25—H25C	109.5
C15—C14—C13	114.1 (2)		
			
C6—C1—C2—O3	−178.3 (2)	C11—C12—C13—C14	−49.1 (3)
C6—C1—C2—C3	−0.1 (4)	C11—C12—C13—C17	72.1 (3)
C1—C2—O3—C7	−0.3 (4)	C11—C12—C13—C16	−168.6 (2)
C3—C2—O3—C7	−178.5 (3)	C12—C13—C14—C15	48.9 (3)
O3—C2—C3—O1	−0.4 (4)	C17—C13—C14—C15	−72.1 (3)
C1—C2—C3—O1	−178.7 (3)	C16—C13—C14—C15	168.0 (3)
O3—C2—C3—C4	178.7 (3)	C11—C10—C15—O4	164.9 (3)
C1—C2—C3—C4	0.4 (4)	C9—C10—C15—O4	−10.3 (4)
O1—C3—C4—O2	0.1 (4)	C11—C10—C15—C14	−13.4 (4)
C2—C3—C4—O2	−179.0 (3)	C9—C10—C15—C14	171.4 (3)
O1—C3—C4—C5	179.8 (3)	C13—C14—C15—O4	162.0 (3)
C2—C3—C4—C5	0.7 (4)	C13—C14—C15—C10	−19.6 (4)
C8—O2—C4—C3	177.3 (3)	C10—C9—C18—C19	−88.5 (3)
C8—O2—C4—C5	−2.4 (5)	C6—C9—C18—C19	136.8 (3)
C3—C4—C5—C6	−2.1 (5)	C10—C9—C18—C23	92.3 (3)
O2—C4—C5—C6	177.6 (3)	C6—C9—C18—C23	−42.5 (3)
C2—C1—C6—C5	−1.2 (4)	C23—C18—C19—O7	−173.2 (3)
C2—C1—C6—C9	−176.9 (2)	C9—C18—C19—O7	7.5 (4)
C4—C5—C6—C1	2.3 (4)	C23—C18—C19—C20	5.2 (4)
C4—C5—C6—C9	177.8 (2)	C9—C18—C19—C20	−174.1 (2)
C1—C6—C9—C10	−177.2 (2)	O7—C19—C20—C21	−151.3 (3)
C5—C6—C9—C10	7.2 (4)	C18—C19—C20—C21	30.2 (4)
C1—C6—C9—C18	−42.5 (3)	C19—C20—C21—C25	67.0 (3)
C5—C6—C9—C18	142.0 (3)	C19—C20—C21—C24	−173.3 (3)
C18—C9—C10—C11	−77.8 (3)	C19—C20—C21—C22	−52.8 (3)
C6—C9—C10—C11	56.1 (3)	C20—C21—C22—C23	45.0 (3)
C18—C9—C10—C15	96.9 (3)	C25—C21—C22—C23	−74.6 (3)
C6—C9—C10—C15	−129.2 (3)	C24—C21—C22—C23	164.9 (3)
C15—C10—C11—O5	−167.2 (2)	C19—C18—C23—O6	165.7 (2)
C9—C10—C11—O5	7.6 (4)	C9—C18—C23—O6	−15.1 (4)
C15—C10—C11—C12	13.6 (4)	C19—C18—C23—C22	−13.8 (4)
C9—C10—C11—C12	−171.5 (2)	C9—C18—C23—C22	165.5 (2)
O5—C11—C12—C13	−159.7 (2)	C21—C22—C23—O6	167.3 (2)
C10—C11—C12—C13	19.5 (4)	C21—C22—C23—C18	−13.2 (4)

### Hydrogen-bond geometry (Å, °)

**Table:**

*D*—H···*A*	*D*—H	H···*A*	*D*···*A*	*D*—H···*A*
O1—H1B···O3	0.85	2.30	2.649 (4)	105
O5—H5B···O6	0.82	1.80	2.604 (3)	166
O7—H7D···O4	0.82	1.84	2.647 (3)	168
C9—H9A···O4	0.98	2.35	2.825 (3)	109
C9—H9A···O7	0.98	2.45	2.865 (4)	105
